# Effect of Training Mode on Post-Exercise Heart Rate Recovery of Trained Cyclists

**DOI:** 10.2478/hukin-2014-0031

**Published:** 2014-07-08

**Authors:** Kelia G. McDonald, Silvie Grote, Todd C. Shoepe

**Affiliations:** 1 Human Performance Laboratory, Department of Health and Human Sciences, Loyola Marymount University, LA, USA; 2 University Honors Program and the Department of Health and Human Sciences, Loyola Marymount University, USA; 3 Visiting Professor, 1 LMU Drive MS 8160, North Hall 206, Los Angeles, CA 90045, USA.; 4 Department of Health and Human Sciences, Loyola Marymount University, LA, USA.

**Keywords:** performance, autonomic shift, aerobic and anaerobic exercise

## Abstract

The sympathetic nervous system dominates the regulation of body functions during exercise. Therefore after exercise, the sympathetic nervous system withdraws and the parasympathetic nervous system helps the body return to a resting state. In the examination of this relationship, the purpose of this study was to compare recovery heart rates (HR) of anaerobically versus aerobically trained cyclists. With all values given as means ± SD, anaerobically trained track cyclists (n=10, age=25.9 ± 6.0 yrs, body mass=82.7 ± 7.1 kg, body fat=10.0 ± 6.3%) and aerobically trained road cyclists (n=15, age=39.9 ± 8.5 yrs, body mass=75.3 ± 9.9 kg, body fat=13.1 ± 4.5%) underwent a maximal oxygen uptake test. Heart rate recovery was examined on a relative basis using heart rate reserve as well as the absolute difference between maximum HR and each of two recovery HRs. The post-exercise change in HR at minute one for the track cyclists and road cyclists respectively were 22 ± 8 bpm and 25 ± 12 bpm. At minute two, the mean drop for track cyclists was significantly (p<0.05) greater than the road cyclists (52 ± 15 bpm and 64 ± 11 bpm). Training mode showed statistically significant effects on the speed of heart rate recovery in trained cyclists. Greater variability in recovery heart rate at minute two versus minute one suggests that the heart rate should be monitored longer than one minute of recovery for a better analysis of post-exercise autonomic shift.

## Introduction

Involuntary body functions are controlled by the autonomic nervous system. The autonomic nervous system has two parts: the sympathetic nervous system which dominates when the body is stressed or excited and the parasympathetic nervous system which helps the body return to and maintain a resting state. During exercise the sympathetic nervous system controls body function, but post-exercise there is a shift in the autonomic nervous system and the parasympathetic system reactivates to return the body to a resting state (Buchheit, Laursen et al., 2007; Buchheit, Papelier et al., 2007). Part of the sympathetic effect during exercise is raising of the heart rate via an epinephrine mediated stimulation of cardiac beta-1 receptors (Borresen and Lambert, 2008) while during rest, the heart rate is lowered to a resting rate via muscarinic activation by acetylcholine through reactivation of the vagal nerve by the parasympathetic nervous system (Borresen and Lambert, 2008). The interaction between parasympathetic and sympathetic activity with regard to controlling the heart rate has been studied using drugs that cause a parasympathetic or sympathetic blockade and observing the effect on the heart rate (Borresen and Lambert, 2008). Such studies have shown that post-exercise decreases in the heart rate are almost exclusively controlled by parasympathetic reactivation as opposed to sympathetic withdrawal (Otsuki et al., 2007) and have confirmed this post-exercise decrease in the heart rate as both a marker of physical fitness and overall cardiovascular health (Buchheit, Papelier et al., 2007).

There are two established, noninvasive methods of detecting sympathetic withdrawal and parasympathetic reactivation in exercising participants: measurement of heart rate recovery (HRRec) and heart rate variability (HRV). The value of HRRec is defined as the rate at which the heart rate decreases to a resting rate after cessation of moderate to heavy exercise (Buchheit, Papelier et al., 2007). This can take from one hour to one day depending on exercise intensity and cardiovascular fitness (Dimkpa, 2009). Despite a range of possible intensities and durations, a drop in the heart rate less than 12 bpm in the first minute of recovery is considered abnormal and indicative of cardiovascular impairment (American College of Sports Medicine, 2009). There is currently no standard in measuring HRRec. Some studies have calculated time constants by fitting HR decay data to mathematical models (Imai et al., 1994; Lamberts et al., 2009) while others look at the difference between the peak heart rate and post-exercise heart rate at one to two minutes after exercise (Heffernan et al., 2007; Shetler et al., 2001). Heart rate variability is defined as the standard deviation of the subjects R-R intervals. High variability is indicative of cardiovascular health whereas low variability is indicative of cardiovascular impairment (Borresen and Lambert, 2008).

While a relationship between HRV and HRRec would be expected due to the fact that they both are measures of a shift in autonomic function, currently no relationship has been observed (Borresen and Lambert, 2008). This suggests that there may be more factors at work in lowering the heart rate post-exercise than parasympathetic alone such as blood metabolites, type of previous exercise training and type of exercise during the assessment (e.g. anaerobic vs. aerobic). Results from Buchheit et al. (2007) seem to confirm different mechanisms as they found that HRRec was faster after endurance exercise trials than after repeated sprint or high intensity exercise trials performed by the same subjects. Subjects’ HRRec and HRV were calculated after moderate continuous exercise, repeated sprint exercise, and high intensity intermittent exercise and they found that parasympathetic reactivation was slower after high intensity and repeated sprint exercise than moderate continuous exercise. Therefore, they suggest that parasympathetic reactivation after sprint exercise is affected more heavily by stress metabolites (i.e. lactate and H+ concentration) and anaerobic contribution than parasympathetic reactivation after aerobic exercise. Furthermore, HRV and HRRec measure different aspects of post-exercise autonomic shift (Heffernan et al., 2007): HRRec being more indicative of vagal tone and HRV being more indicative of parasympathetic activation (Borresen and Lambert, 2008). In other work, Heffernan (2007) looked at changes in HRV and HRRec after short-term resistance training protocols and he found no change in HRV, but did see change in HRRec. Both variables are valuable to athletes because they are good markers of overall fitness and training status, including overtraining (Lamberts et al., 2009). However, HRRec is the most easily applicable method for physical performance training and conditioning, as it requires no special equipment.

Results of previous studies suggest that autonomic adaptation to exercise varies depending on the training mode and type of exercise performed. Multiple studies have shown that HRRec improves with training in both sedentary and already trained subjects (Heffernan et al., 2007; Kestin, 1993; Lamberts et al., 2009; Otsuki et al., 2007). Fourteen well-trained cyclists participated in a 4-week high-intensity training program and showed a significant increase in HRRec at 60 s after cessation of a maximal exercise test (Lamberts et al., 2009; Otsuki et al., 2007). The time constant of post-exercise heart rate decay, another method of measuring vagally mediated post-exercise HRRec, was shown to be decreased in strength trained and endurance trained subjects when compared to sedentary subjects. However, the decrease in decay did not display a training specific response as no significant difference was observed between the two trained groups (Otsuki et al., 2007). Other similar studies have observed a similar improvement in post-exercise HRRec after resistance training and further suggest that the mechanisms of post-exercise HRRec may be slightly different in strength (anaerobic) and endurance (aerobic) trained athletes (Heffernan et al., 2007). As such, the purpose of the current study was to compare the effect of a continuous training mode on heart rate recovery of trained track (anaerobic) and road (aerobic) cyclists at one and two minutes post-exercise.

## Material and Methods

### Participants

Ten track cyclists (1 female, 9 male) and 15 road cyclists (4 female, 11 male) were recruited to participate in the study. Most track cyclists were solicited via a flyer that was posted at a training facility, while the road cyclists and remaining track cyclists were recruited from a performance testing medical office. All participants were amateur athletes who trained more than six hours per week in their respective training modes for at least 2 years. Cyclists trained at the following intensities based on the respiratory exchange ratio (RER = CO2/ O2) (Beam and Adams, 2011): 65–80%, 80–95%, 95–105%, and 105% to max, following training protocols of their respective coaches and time of the season. All subjects gave written informed consent and completed a Physical Activity Readiness Questionnaire (e.g. PAR-Q) before inclusion in this study to ensure that they were free of any injury or medical condition that may have affected safety or performance (American College of Sports Medicine, 2009). All procedures were approved by the Institutional Review for the Protection of Human Subjects of the Loyola Marymount University prior to the initiation of the study and a written informed consent form was obtained from each participant.

### Measures

All data was collected during a one-day session, which consisted (in order) of resting values, maximal exercise testing, and recovery variables. Testing to measure maximal oxygen uptake (VO2max), the maximum heart rate (HRMax), and recovery heart rates (HRMin1 and HRMin2) took place in the Human Performance Laboratory at the Loyola Marymount University using an Ergomedic 894E Peak Bike (Monark EB; Varberg, Sweden). Respiratory gas exchanges were measured using a Vmax Spectra 29 Metabolic Cart (SensorMedics; Homestead, FL) and calibrated for volume, flow, and gas concentrations prior to each test. A 12-lead electrocardiogram (ECG) during rest and exercise was obtained to monitor the heart rate and ensure safety respectively. Seven-site skinfold procedures (Jackson et al., 1980) were used with Lange calipers (Beta Technology, Santa Cruz, CA) to determine body density, then percent body fat was estimated using the Siri equation (Siri, 1956).

### Procedures

After a supine resting 12-lead ECG and a resting heart rate were obtained, participants were permitted to warm up as long as necessary. These athletes were highly trained athletes so they had established individualized warm-up procedures in place. Following an average warm up time of 10 minutes, they were fitted with a respiratory valve and nose clip immediately prior to the start of the test. During the test, the subject began pedaling at a comfortable pace where for these participants, rpms ranged between 75–90 throughout the test. The power was increased in 50 watt increments every two minutes until the end of the test and the following physiological variables were recorded every 10 seconds: oxygen uptake, respiratory exchange ratio, heart rate. Power and rate of perceived exhaustion using a Borg scale (Borg, 1982) were also collected manually every 2 minutes. The test was concluded according to the criteria of maximum exercise test termination set out by the ACSM (Beam and Adams, 2011). This is when two out of four of the following criteria were met: (1) heart rate came within 10–12 beats of age predicted maximum, (2) there was a plateau in oxygen uptake even with increased workload, (3) respiratory exchange ratio became greater than 1.15, or (4) subject requested to stop. Upon completion of the test protocol, resistance was reduced to pre-test wattage, and the subjects were allowed to pedal lightly for the first minute as recommended by the ACSM’s Guidelines for Exercise Testing and Prescription (2009) but were asked to sit without speaking or moving for the second minute of recovery. The cyclists’ short-term HRRec variables were calculated as the maximum heart rate during the test minus the heart rate at one minute (HRmin1) and two minutes (HRmin2). Relative change in heart rate was calculated as maximum heart rate minus HRmin1 or HRmin2 divided by HRmax minus HRresting.

### Statistical Analysis

Participant descriptive data are shown in Table 1 and reported as mean ± SD. Between groups differences were determined by t-tests for height, body mass, age, %BF, VO2max, and differences between training groups. The Levene’s test for homogeneity of variance was performed on all dependent variables which were found to be normal. A one-way ANOVA test was conducted to evaluate differences between a relative change in the heart rate at minutes one and two (calculated as the difference between HRmax and the recovery heart rate divided by the difference between HRmax and HRresting) and the drop in beats per minute (bpm) between the max HR and the recovery heart rate at minutes one and two. Statistical significance for all tests was set at p < 0.05. Subsequent ANOVA tests determined that there was no significant difference in HRRec when grouped by gender and that maximal oxygen uptake was not significantly different between training groups. Therefore, gender data remained pooled based on the training type for the analysis of the between-groups difference. Statistical testing was carried out using the Statistical Package for the Social Sciences version 18.0 for Mac (PASW, Chicago, IL).

## Results

There was a significant difference between the ages of participants in the groups and Pearson correlations revealed a low but statistically not significant relationship between age and HRRec (r = 0.23, p = 0.26). Testing variables including those of recovery are included in Table 2. The mean post-exercise change in the heart rate at minute one was 22 ± 8 bpm for the track cyclists and 25 ± 12 bpm for the road cyclists. At minute two, the track cyclists had a mean drop of 52 ± 15 bpm and the road cyclists had a mean drop of 64 ± 11 bpm. The relative drop in the HR was also greater for the road cyclists at each time point than the track cyclists (Minute 1: 19% for road, 16% for track; Minute 2: 48% for track, 40% for road). The mean drop in the heart rate when calculated as a percent or a difference in beats per minute was significantly different between the two training groups at minute two (p=0.33, p = 0.03) but not at minute one (p=0.48, p=0.44).

## Discussion

The main purpose of this study was to compare the rate of post-exercise recovery of trained track and road cyclists by measuring their recovery heart rates at one and two minutes after maximal exercise testing. The aerobically trained road cyclists showed faster HR_Rec_ than the anaerobically trained track cyclists at both one and two minutes of recovery, with a statistically significant difference between the two groups at minute two (track 40%, road 48%). These results were expected as aerobic endurance training had shown to improve other variables indicative of cardiovascular health such as left ventricular wall thickness (Otsuki et al., 2007) and relative maximal oxygen uptake. However, these differences in HR_Rec_ between the two groups were only significant at minute two.

This differs somewhat from the findings of previous studies. A study conducted by Otsuki et al. (2007) also found differences in recovery heart rates of endurance and strength athletes, but these differences were not significant. However, one marked difference between the current study and that of Otsuki et al. (2007) is that they used mathematical modeling to describe heart rate recovery during the first 30 seconds of recovery. They concluded that post-exercise heart rate recovery may be carried out differently in strength and endurance trained athletes. Heffernan et al. (2007) made a similar suggestion that resistance exercise seems to increase vagal tone (defined as release of acetylcholine by the vagus nerve on the heart’s autorhythmic cells and increased receptor number/sensitivity) more than parasympathetic modulation. This suggests that it would be most useful to use several different variables to study the effect of the training mode on overall post-exercise autonomic shift to account for specific influence of different training modes. Further studies are also needed to better define the specific implications of variables such as HR_Rec_ and HRV as individual variables may be more meaningful to specific athletic and clinical populations.

We observed that the change in the HR at minute two, whether calculated as a simple difference or as a relative change, was more indicative of overall heart rate recovery ability in highly trained athletes than minute one. Although the difference at this time point was not statistically significant, there was greater variability between the two groups than when considering the HR at minute one. While there is no standard for measuring HR_Rec_, this suggests that the recovery HR should be monitored past minute one for better understanding of overall autonomic shift, at least for athletic applications. In contrast, Watanabe et al. (2001) found that one minute heart rate recovery was more statistically related to all-cause mortality than two or three minute heart rate recovery when testing patients referred for stress testing. This suggests that the time points or measures of HR_Rec_ most useful for clinical applications may be different from time points or measurements useful to studies looking to measure training status or fitness.

In conclusion, the recovery HR has been used frequently in monitoring of overall fitness and training status (Lamberts et al., 2009), where a faster recovery rate indicates optimal fitness and absence of overtraining. From a practical standpoint, coaches and athletes can use postexercise change in the HR as an indicator of overtraining, keeping in mind that varied modes of training elicit different recovery rates. As more studies examine the exact methods of post-exercise autonomic shift and the variables that describe it, it is clear that it is a very complex system. The HR_Rec_ and other related variables have obvious value in both clinical and athletic applications, but more research is needed to clearly define what part of post-exercise recovery such variables describe. In athletic applications, this would allow coaches and athletes to use these measures of post-exercise autonomic shift to better monitor specific aspects of training, including overtraining, and recovery as it relates to the athletes’ training mode.

## Figures and Tables

**Table 1:** Selected characteristics of study participants.

	**Track (*n*=11)**	**Road (*n*=15)**
Age (years)	25.9 ± 6.0	39.9 ± 8.5
Gender	2 F, 9 M	4 F, 11 M
Body mass (kg)	82.7 ± 7.1^*^	75.3 ± 9.9
% Body Fat	10.0 ± 6.3^*^	13.1 ± 4.5
Resting HR (bpm)	56 ± 6.9	52 ± 7.9
Heart Rate Reserve (bpm)	133 ± 9.9	134 ± 7.8

* Denotes statistically different than road (p<0.05). bpm = heart beats per minute.

All values are reported as mean ± SD.

**Table 2:** Results of maximal oxygen uptake test.

	**Track (*n*=11)**	**Road (*n*=15)**
Peak Power (W)	352.3 ± 48.5	372.7 ± 69.6
Relative Peak Power (W·kg^−1^)	4.4 ± 0.6	5.0 ± 0.7
Standard VO_2max_ (mL·kg^−1^·min^−1^)	54.9 ± 5.4	53.4 ± 7.0
HR_Rec_ Minute 1 (bpm)	22 ± 8	25 ± 12
HR_Rec_ Minute 2 (bpm), %	53^*^ ± 15	64 ± 11
Percent HR_Rec_ Minute 1	16%	19%
Percent HR_Rec_ Minute 2	40%^*^	48%

* Denotes statistically different than road (p<0.05).

All values are reported as mean ± SD.

W = watts, mL = milliliters, HR_Rec_ = heart rate recovery, VO_2max_ = maximal oxygen consumption, kg = kilograms, and bpm = heart beats per minute.
